# 3-M syndrome associated with growth hormone deficiency: 18 year follow-up of a patient

**DOI:** 10.1186/1824-7288-39-21

**Published:** 2013-03-21

**Authors:** Cristina Meazza, Ekkehard Lausch, Sara Pagani, Elena Bozzola, Valeria Calcaterra, Andrea Superti-Furga, Margherita Silengo, Mauro Bozzola

**Affiliations:** 1 Department of Internal Medicine and Therapeutics, University of Pavia, Fondazione IRCCS Policlinico San Matteo, Piazzale C. Golgi 2, Pavia 27100, Italy; 2 Division of Paediatric Genetics, Centre for Paediatrics and Adolescent Medicine, University of Freiburg, Freiburg, Germany; 3 U.O. di Pediatria Generale e Malattie Infettive, Ospedale Pediatrico Bambino Gesù, Roma, Italy; 4 University of Lausanne, Centre Hospitalier Universitaire Vaudois, Lausanne, Switzerland; 5 Department of Paediatrics, University of Torino, Torino, Italy

**Keywords:** 3-M syndrome, Growth hormone deficiency, *CUL-7* gene mutation

## Abstract

3-M syndrome is a rare autosomal recessive disorder that causes short stature, unusual facial features and skeletal abnormalities. Mutations in the *CUL7*, *OBSL1* and *CCDC8* genes could be responsible for 3-M syndrome.

Here we describe the growth and evolution of dismorphic features of an Italian boy with 3-M syndrome and growth hormone deficiency (GHD) from birth until adulthood. He was born full term with a very low birth weight (2400 g=−3.36 standard deviation score, SDS) and length (40.0 cm =−6.53 SDS). At birth he presented with a broad, fleshy nose with anteverted nostrils, thick and patulous lips, a square chin, curvilinear shaped eyebrows without synophrys, short thorax and long slender bones. Then, during childhood tall vertebral bodies, hip dislocation, transverse chest groove, winged scapulae and hyperextensible joints became more evident and the diagnosis of 3-M syndrome was made; this was also confirmed by the finding of a homozygous deletion in exon 18 of the *CUL7* gene, which has not been previously described.

The patient also exhibited severe GHD (GH <5 ng/ml) and from the age of 18 months was treated with rhGH. Notwithstanding the early start of therapy and good compliance, his growth rate was always very low, except for the first two years of treatment and he achieved a final height of 132 cm (−6.42 SDS).

## Background

3-M syndrome (OMIM273750) is a rare autosomal recessive disorder that causes short stature, unusual facial features and skeletal abnormalities [1]. The name of this condition comes from the initials of the three researchers (Miller, McKusik and Malvaux) who first identified it. Individuals affected by 3-M syndrome have intrauterine growth retardation (IUGR) and postnatal growth retardation, worsened also by feeding difficulties during the first years of life. Affected patients usually have a triangle-shaped face with a pointed chin, large ears, full eyebrows, an upturned nose with a fleshy tip, long philtrum, and a prominent mouth with full lips. They may also exhibit prominent shoulder blades and square shoulders, exaggerated curvature of the lower back (hyperlordosis), pectus carinatum or excavatum, prominent heels and other skeletal abnormalities which are apparent in X-ray images [2].

Mutations in the *CUL7* gene cause 3-M syndrome, although genetic heterogeneity has been reported [3,4] involving two other causative genes, *OBSL1* and *CCDC8*[5-7]. Since CUL7 is involved in chondrocyte growth and proliferation, in 3-M syndrome, reduced cell mitosis during the early gestation period could be the cause of retarded growth. In particular, these mutations disrupt the ability of the protein cullin-7 to bring together the components of the ubiquitine-proteasome system which is involved in the degradation of unwanted proteins. Therefore, impaired ubiquitination may have a role in the pathogenesis of IUGR in humans.

Here, we describe a patient with 3-M syndrome and GH deficiency, who was followed from the age of two years to adulthood. Even though rhGH therapy was started early in infancy (18 months of age) with good compliance, no catch-up growth was observed.

## Case presentation

**Figure 1:**
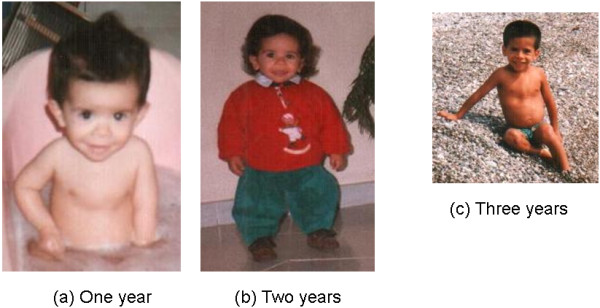
The patient at one (a), two (b) and three (c) years of age with typical 3-M syndrome facial features including frontal bossing, relatively increased head circumference, triangular face, hypoplastic midface, low nasal bridge, depressed nasal root, fleshy upturned nose, long philtrum, full lips, and pointed, prominent chin.

**Figure 2:**
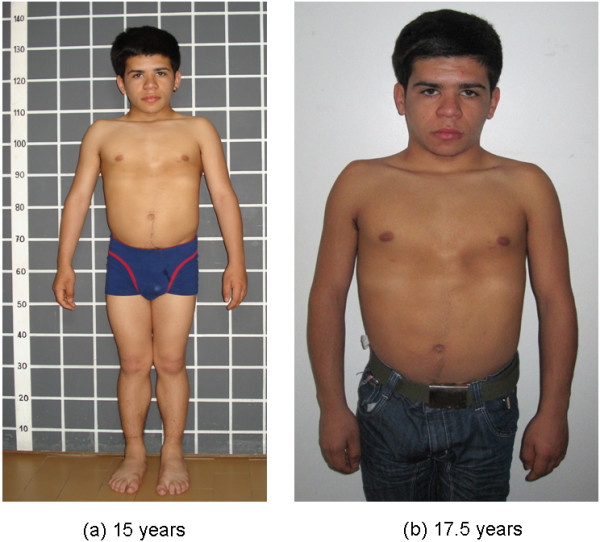
Patient at 15 (a) and 17.5 (b) years showing facial dismorphism, short stature, broad thorax, sternum carinatum.

**Figure 3:**
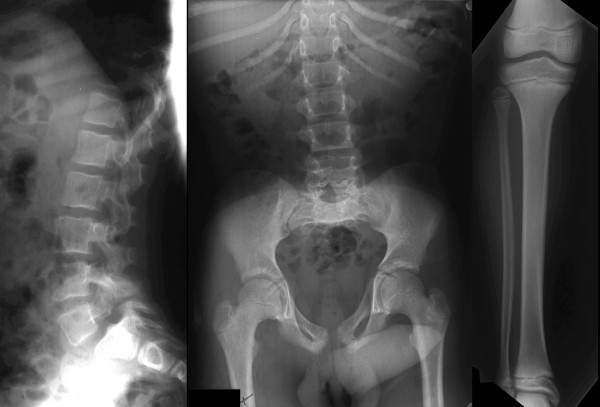
**Radiographic features of the adult 3-M syndrome patient.** On the lateral lumbar spinal radiograph (left panel), vertebral bodies are relatively tall and short. Lumbar hyperlordosis is caused by the short femoral necks. In the central panel, note the short femoral necks with anterior rotation of the pelvis (small foramina obturatoria). The ribs and the visible part of the femoral shafts are slender. The distance between the vertebral pedicles remains constant from L1 to L5. The tibia and fibula (right panel) are relatively inconspicuous. The radiographic features fitting with the diagnosis of 3-M are the tall and narrow vertebral bodies and slender tubular bones; other features of a chondrodysplasia, such as platyspondily or metaphyseal and epiphyseal changes, are typically absent.

An Italian boy born after 42 weeks gestation complicated by partial detachment of the placenta was evaluated at our institute from the age of two months. Birth length was 40.0 cm (−6.53 standard deviation score, SDS) and birth weight was 2,400 g (−3.36 SDS). His target height was 165 cm (−1.28 SDS). At two months he showed some dismorphic features: broad and fleshy nose with anteverted nostrils, thick and patulous lips, square chin, curvilinear shaped eyebrows without synophrys, short thorax, distended abdomen, flat feet, long slender bones and ribs which contrasted with the child’s stocky appearance (Figure 1a and 1b). Only at 3 years did the patient exhibit a large head circumference (the head was disproportionately large in comparison with the body), prominent forehead, anteverted nares, round face, frontal bossing and short broad neck with prominent trapezius muscles, tall vertebral bodies, and hip dislocation (Figure 1c). From the age of 7–8 years triangular-shaped face, frontal bossing, transverse chest groove, winged scapulae and hyperextensible joints became very evident (Figures 1a and 2b and Figure 3). Since radiological findings suggested hypochondroplasia, a first genetic analysis of exon 13 of the *FGFR-3* gene was performed. However, no mutations associated with hypochondroplasia were found. Finally, the diagnosis of 3-M syndrome was made, confirmed by the analysis of the *CUL7* gene. In fact, an homozygous deletion of the 10 bp TTGGCTACCC (c.3388_3397del10) in exon 18 of the *CUL7* gene was found. This variation found is not an annotated SNP and has not been previously described. The deletion predicts a frameshift and a preterminal stop codon (p.Leu1130GlyfsX8) resulting in a truncated cullin 7 protein. The homozygous 10 bp deletion is, therefore, very likely the cause of the patient’s type 1 3-M syndrome. His parents, his younger brother and his older sister are all unaffected heterozygous carriers of the deletion. The sister is healthy, without dismorphic features and a final height around the 10^th^ percentile. On the contrary, the brother shows some facial dismorphisms, a psychomotor delay and cardiological defects, not related to 3-M syndrome.

**Figure 4:**
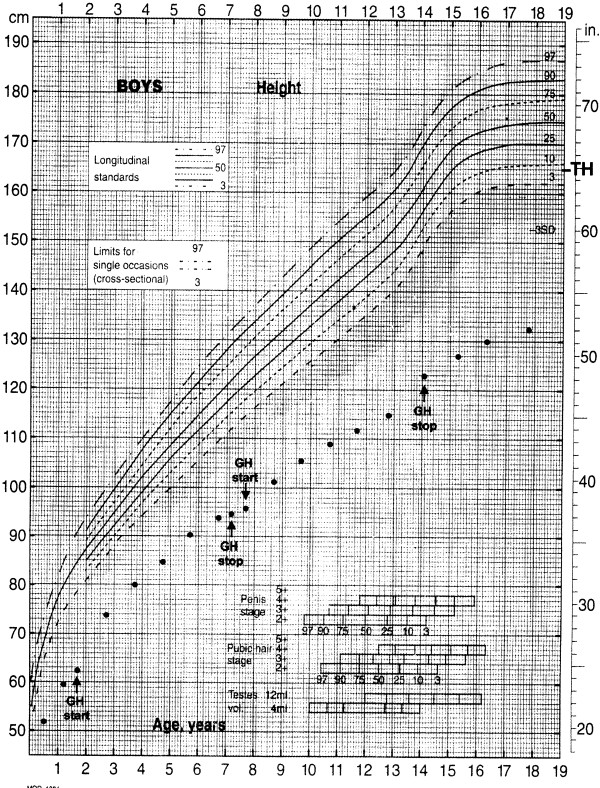
**Growth chart for height of the patient from birth until adult age.** TH=target height.

At the age of 18 months he underwent an endocrinological evaluation since his height was 64 cm (−6.57 SDS) and bone age was less than 12 months; a diagnosis of growth hormone deficiency (GHD) was made (GH peak after arginine: 0.93 ng/ml; GH peak after glucagon: 4.19 ng/ml; cut-off 10 ng/ml). Thyroid and cortisol functions were normal. Celiac disease was also excluded. rhGH therapy was started at the dose of 0.25 mg/kg/week and his height was measured every six months (Figure 4); after the first year of treatment the growth rate was 10.1 cm (1.63 SDS) and after the second year it was 6 cm (−0.27 SDS). At the age of 7.1 years, his growth failure became more evident (height 94.3 cm=−4.52 SDS, bone age of 5 years, growth rate 1.4 cm/year=−4.46 SDS) and rhGH therapy was discontinued. After some months, GH secretion was re-evaluated and partial GHD was confirmed (GH peak after arginine: 4.0 ng/ml; GH peak after glucagon: 7.15 ng/ml), therefore rhGH therapy was restarted until the patient was 14.3 years-old, when it was definitively interrupted. His height was 122.5 cm (−4.25 SDS) with a growth rate of 4.2 cm/year (−0.42 SDS). His bone age was 12.5 years. The insulin-like growth factor-I (IGF-I) generation test showed an increase from −0.5 SDS to 0.5 SDS and GH secretion after arginine was 17.3 ng/ml. His final stature at 18 years is 132 cm (−6.42 SDS).

We observed a slight delay in pubertal development; at the age of 13.8 years he showed reduced luteinizing hormone (LH) levels (2.0 IU/L; normal values 6–30 IU/L) and a gonadal volume of about 6 ml. However, at 18 years his gonadal volume was within the normal range (15–20 ml).

## Discussion

3-M dwarfism is a clinical entity which is not often recognized in childhood. Here, we describe a patient with 3-M syndrome associated with GHD. The child was very small at birth and severe growth restriction continued in the post-natal period and throughout childhood. In fact, his final stature is −6.42 SDS. In our patient, skeletal changes typical of 3-M syndrome, such as long slender bones, tall vertebral bodies, triangular-shaped face with square chin, short thorax, transverse chest groove and winged scapulae were not present at birth, but manifested in the course of childhood. Therefore, the diagnosis of 3-M syndrome was made only when the patient was 17 year-old, after having investigated other potential syndromes such as hypocondroplasia. However, an early diagnosis is important for familial genetic familiar counselling since 3-M syndrome has an autosomal recessive mode of inheritance. It is important to closely follow patients with syndromic features and perform genetic analyses as the dismorphic features become apparent.

3-M dwarfism belongs to a group of intrauterine growth retardation-malformation syndromes. Therefore, differentiation from Russell-Silver syndrome is difficult because of the phenotypic variability of the latter. Body asymmetry is not present in 3-M syndrome, while short stature with prenatal onset and a small triangular face are found in both syndromes. However, 3-M subjects are shorter than those with Russell-Silver syndrome [8,9].

The most striking feature of 3-M syndrome is severe growth retardation. According to other cases reported in the literature, the present patient achieved a height between 4 and 6 SD below the mean [10]. Since our patient also exhibited severe GHD, rhGH therapy was started. To the best of our knowledge only one other case of partial GH deficiency in a 3-M syndrome patient has been described [1]. However, in the six cases described by van der Wal et al. [10], five patients were treated with rhGH independently from normal GH secretion, but only the two patients who started GH treatment at a prepubertal stage showed a good response and their adult height was well above the final height of the other three cases. On the other hand, Russell-Silver syndrome patients benefit from GH supplementation even in the absence of GHD [11] and show significant growth acceleration and improved final height, even when GH therapy is initiated later in life [12]. In our patient, even though rhGH therapy was started early in infancy (18 months of age) with good compliance, no catch-up growth was observed. His slow growth continued throughout childhood and adolescence leading to a final height of 132 cm, well below his target height. Our patient’s blunted response to rhGH therapy may be due to the presence of the mutation in the *CUL7* gene which may strongly affect GH effects on longitudinal growth. In fact, it has been reported that subjects with *CUL7* mutations are significantly shorter than those with *OBSL1* or *CCDC8* mutations [7].

Finally, we observed a rather normal sexual development confirming that male gonadal dysfunction is not a concomitant feature of 3-M syndrome, as also reported in previous studies [1].

In conclusion, there are many typical features of 3-M syndrome, but, apart from a very severe growth retardation, patients may not exhibit these from birth. Many other syndromes with short stature should be considered when making a differential diagnosis of 3-M syndrome, i.e. Silver-Russel syndrome, Bloom syndrome, Dubowitz syndrome, Rubistein-Taybi syndrome, Floating-Harbor syndrome, Mulibrey nanism and fetal alcohol syndrome, but the most difficult to differentiate is Silver-Russel syndrome. However, during childhood, subjects with 3-M syndrome develop striking radiological abnormalities, making the diagnosis possible. Finally, the diagnosis should be confirmed by the presence of mutations in the *CUL7* gene, which account for about 80% of 3-M syndrome patients [4].

## Consent

Written informed consent was obtained from patient’s parents for publication of his Case report and any accompanying images. A copy of the written consent is available for review by the Editor-in-Chief of this journal.

## Abbreviations

GH: Growth hormone; GHD: Growth hormone deficiency; SDS: Standard deviation score; IUGR: Intrauterine growth retardation; IGF-I: Insulin-like growth factor-I; LH: Luteinizing hormone.

## Competing interests

The authors declare that they have no competing interests.

## Authors’ contributions

CM acquired the data of the patient and drafted the manuscript. EL carried out the genetic molecular studies. SP helped to acquire the data and draft the manuscript. EB helped to draft the manuscript. VC analyzed the data and helped to draft the manuscript. ASF supervised the genetic analysis and critically revised the manuscript. MS participated to the diagnosis and critically revised the manuscript. MB made a contribution in conception of the manuscript and helped to draft the manuscript. All the authors gave final approval of the version to be published. All authors read and approved the final manuscript.
